# Measuring cell deformation by microfluidics

**DOI:** 10.3389/fbioe.2023.1214544

**Published:** 2023-06-27

**Authors:** Ling An, Fenglong Ji, Enming Zhao, Yi Liu, Yaling Liu

**Affiliations:** ^1^ School of Engineering, Dali University, Dali, Yunnan, China; ^2^ School of Textile Materials and Engineering, Wuyi University, Jiangmen, Guangdong, China; ^3^ Department of Bioengineering, Lehigh University, Bethlehem, PA, United States; ^4^ Department of Mechanical Engineering and Mechanics, Lehigh University, Bethlehem, PA, United States

**Keywords:** microfluidics, cell deformation, cell imaging, high-throughput analysis, cell mechanical characterization

## Abstract

Microfluidics is an increasingly popular method for studying cell deformation, with various applications in fields such as cell biology, biophysics, and medical research. Characterizing cell deformation offers insights into fundamental cell processes, such as migration, division, and signaling. This review summarizes recent advances in microfluidic techniques for measuring cellular deformation, including the different types of microfluidic devices and methods used to induce cell deformation. Recent applications of microfluidics-based approaches for studying cell deformation are highlighted. Compared to traditional methods, microfluidic chips can control the direction and velocity of cell flow by establishing microfluidic channels and microcolumn arrays, enabling the measurement of cell shape changes. Overall, microfluidics-based approaches provide a powerful platform for studying cell deformation. It is expected that future developments will lead to more intelligent and diverse microfluidic chips, further promoting the application of microfluidics-based methods in biomedical research, providing more effective tools for disease diagnosis, drug screening, and treatment.

## 1 Introduction

Cell deformation refers to a change in cell shape in response to external stimuli or internal changes. This process is crucial for various cellular functions, such as migration, division, and mechanotransduction (Tomaiuolo et al., 2009). The ability to measure cellular deformation provides important insights into cellular biomechanical properties that have been shown to be closely linked to cellular function and disease development. For example, in cancer, the mechanical properties of cells are altered due to various genetic and environmental factors, leading to changes in cell migration, invasion, and metastasis (Lv et al., 2021), (Lei et al., 2021), (Tsai et al., 2014). Therefore, measuring cellular deformation in cancer cells has become an important tool for understanding the underlying mechanisms of cancer development and developing new diagnostic and therapeutic approaches (Lee and Lim, 2007), (Gustafson et al., 2015). In addition to cancer, cellular deformation is also relevant to various other fields such as immunology, stem cell research, and tissue engineering. In immunology, for example, the deformation of immune cells has been shown to be important for their interaction with pathogens and other cells in the immune system. In stem cell research, the mechanical properties of stem cells can provide information about their differentiation potential and ability to integrate into tissues. In tissue engineering, cell deformation in response to mechanical cues is critical for the design and fabrication of functional tissue structures (Tsai et al., 2015). In addition, RBC deformability has been linked to human disorders and can be assessed based on RBC velocity using a microfluidic constriction (Tsai et al., 2016). Given the importance of cell deformation in various fields, it is crucial to develop reliable and accurate methods to measure cell deformation. Microfluidics-based methods have emerged as promising tools for this purpose, providing high-throughput, accurate and controlled measurements of cell deformation.

In recent years, the fields of microelectronics, micromechanics, biological engineering, and nanotechnology have combined to form the interdisciplinary field of microfluidics. With microfluidics, precise distribution, mixing and the reaction of tiny amounts of liquids can be achieved, as well as the localization, capture, manipulation and analysis of microparticles and cells (Sackmann et al., 2014). As one of the most advanced technologies in the world, microfluidics is widely used for applications in biology, medicine and chemistry (Sun et al., 2020). Microfluidics has shown great potential for technologies such as cell isolation and drug screening, and microfluidics has gained popularity as a potential replacement for conventional experimental methods because of the features such as quick sample processing and precise fluid control in assays (Beebe et al., 2002). Compared with traditional methods, microfluidic technology has many advantages in measuring cell deformation. Table 1 summarizes the advantages of microfluidic technology and traditional methods in measuring cell deformation.

**TABLE 1:** Comparison of advantages of microfluidic method and traditional method for cell deformation measurement.

Parameter	Microfluidic method	Traditional method
Measurement speed	High	Low
	Multiple cells can be processed simultaneously on a single chip, improving measurement speed	Each cell is measured independently, and it takes more time to measure multiple cells, thus the measurement is slow
Measurement accuracy	High	Low
	Accurately control the stress and shear force of cells to improve the measurement accuracy	The external force and deformation degree of cells cannot be controlled and regulated accurately, thus limiting the improvement of measurement accuracy
Sample consumption	Low	High
	Measurements can be made using only a few hundred to a few thousand cells, reducing the consumption of biological samples	Multiple operations are required to obtain reliable data, resulting in a large number of sample consumption
Experimental cost	Low	High
	The required equipment and materials are relatively simple, common and inexpensive, and do not require high equipment investment and maintenance costs	Need dedicated equipment
Degree of experimental automation	High	Low
	The automation device carries out experimental operation and computer program control, thus achieving a high degree of experimental automation	Manual operation required
Controllability	High	Low
	Precise fluid control to regulate fluid velocity, pressure, and direction, thereby precisely controlling the external forces and strains on the cell	Limited by experimental conditions and devices, precise force and strain control cannot be achieved

Microfluidics-based methods are used to measure cell deformation by studying the response of cells to physical or chemical stimuli using microfluidic devices (Hou et al., 2009). These devices allow the manipulation of fluids to create precise microenvironments for controlled observation of cellular behavior. Microfluidic devices can generate highly controllable fluid flow and shear stress, which can be tailored to study cell deformation in response to different mechanical or chemical cues. They are usually made of biocompatible materials such as PDMS and can be easily combined with various imaging modalities such as phase contrast, confocal and fluorescence microscopy (Yager et al., 2006). Microchannels are a common strategy used in measuring cell deformation using microfluidics. Cells are confined in narrow channels where controlled flow or pressure can be applied. Changes in cell shape or displacement of cell markers in response to mechanical or chemical stimuli are measured to quantify cell deformation. Other microfluidics-based techniques for measuring cell deformation include droplet microfluidics, where cells are encapsulated in droplets and subjected to mechanical or chemical forces, and microcolumn arrays, which allow the measurement of cellular forces through the deformation of microcolumns (Whitesides, 2006). These techniques provide a powerful tool for studying the mechanical and biochemical responses of cells to various stimuli with high spatial and temporal resolution. They can be customized to study a wide range of cellular processes and functions (Tsai et al., 2016).

This paper reviewed the recent advancements in microfluidics for measuring cell deformation while discussing different types of microfluidic devices and outlining different methods for inducing cell deformation in these devices. The paper reviewed the three methods reported previously for measuring cell deformation by microfluidics. One method is to inject single or multiple cells into a microfluidic channel and sense the deformation of the cells in the liquid by applying pressure. The optical method uses optical tweezers and optical stretching to identify the deformability of the cells. The latest developed method is real-time deformability cytometry (RT-DC), which uses optical microscopy with data analysis software to identify and analyze cells in real time and measure their deformation. In summary, microfluidics can provide a reproducible and controlled method to measure cell deformation, thus increasing the understanding of cell biomechanical behavior and providing a new means for biomedical research and clinical applications.

## 2 Principles of microfluidics-based cell deformation measurement

### 2.1 Different types of microfluidic devices

Passive microfluidic devices use physical properties such as surface tension, capillary action, and gravity to manipulate fluid flow, which means they do not require external energy input to control the flow. Examples of passive microfluidic devices include microchannels, microvalves, and microfilters (Niculescu et al., 2021), (Scott and Ali, 2021). These devices can achieve simple operations such as fluid transport, separation, and mixing, but they have limited functionality for more complex operations such as sample manipulation, cell sorting, and drug screening. (Catarino et al., 2019). Active microfluidic devices, on the other hand, use external energy sources to drive fluid flow and manipulate samples. These devices can be controlled precisely and dynamically using electric, magnetic, thermal, or acoustic fields. Examples of active microfluidic devices include microfluidic pumps, electrophoresis chips, and dielectrophoresis-based devices. These devices can achieve complex operations such as sample manipulation, cell sorting, and high-throughput drug screening, but they require more complex fabrication and control techniques, and they are typically more expensive. (Battat et al., 2022). Hybrid microfluidic devices combine both passive and active control methods to achieve more functionalities. These devices can use passive methods for basic fluid transport and mixing, while active methods can be used for more complex operations such as cell sorting and sample manipulation. Hybrid microfluidic devices can also be designed to integrate multiple functions, such as sensing and analysis, into a single device. Hybrid microfluidic devices offer a versatile platform for a variety of applications, including biomedical research, clinical diagnostics, and drug discovery.

Passive microfluidic devices are often limited in their functionality and throughput, but they are useful for applications where precise fluid control is not required and where the sample volume is limited. They are also useful for applications where low-cost and easy fabrication are desired, such as in point-of-care diagnostics or portable lab-on-a-chip devices (Narayanamurthy et al., 2020). However, for more complex applications that require higher throughput and more precise fluid control, active microfluidic devices are preferred. These devices require external energy sources to drive fluid flow and can achieve more complex operations such as mixing, separation, and sorting. Examples of active microfluidic devices include electrokinetically-driven microfluidic devices, where electric fields are used to drive fluid flow, and microfluidic devices that utilize acoustic waves or magnetic fields to manipulate fluids and particles (Guzzi et al., 2020). Active microfluidic devices can achieve faster fluid flow rates, higher throughput, and more precise fluid control, but they are also more complex to fabricate and require more sophisticated control systems (Wang et al., 2015), (Herbig et al., 2018).

Active microfluidic devices employ external excitation to regulate the flow behavior of liquids in microfluidic systems. Dielectrophoretic force-driven, magnetic field force-driven, and ultrasonic wave-driven microfluidic devices are commonly used as external excitation for active microfluidic devices. Microfluidic chip-based dielectric electrophoresis-based separation and collection techniques have been extensively used in the field of blood cell separation because they are label-free, do not require surface modification, and are extremely selective (Teng et al., 2017), (Teng et al., 2018). For instance, Nascimento et al. (2008) separated and collected parasite-infected red blood cells from healthy red blood cells using microfluidic dielectrophoresis. The magnetic field force can lead the target blood cells to deviate from their initial path, so blood cells with varying sizes and magnetization rates will be subjected to varied magnetic field forces when flowing through the gradient magnetic field, resulting in separation and collection (Karabacak et al., 2014). Zhao et al. (2017) created two microfluidic devices to use varying magnetic fluid stresses on various cell types to separate circulating tumor cells from leukocytes and erythrocytes. Moreover, erythrocyte suspension in the magnetic medium can be measured using the magnetic fluid method, allowing researchers to evaluate the erythrocytes’ deformation capabilities (Tasoglu et al., 2015). The principle of the ultrasonic drive is to apply a certain angle of sound waves to the fluid through the microchannel, and the pressure generated can push the particles suspended in the liquid to move, compared with normal blood cells, the sound wave has a greater thrust on cancer cells, so tumor cells can be pushed into a separate microchannel. For example, Ding et al. (2014) designed a microfluidic device with surface sound waves to achieve the separation and capture of circulating tumor cells, and used this method to successfully detect extremely rare tumor cells from the blood of breast cancer patients (Li et al., 2015). Each type of microfluidic device has its own advantages and limitations respectively. For instance, one microfluidic device may have the advantages of high speed, high precision, and simple operation on one hand. On the other hand it may also require complex manufacturing processes, or exhibit limited control accuracy due to various external factors.

Due to the limitations of individual passive or active techniques, many microfluidic tools have been developed to combine the advantages of both. These tools have been used to study various aspects of cell biology, such as cell culture, manipulation, and analysis (Salafi et al., 2019). The most commonly used microfluidic tools for cell research include devices for cell culture, manipulation, analysis, and single-cell analysis (Lenshof and Laurell, 2010). These devices typically use active elements to control fluid velocity and direction, while passive elements rely on the geometric shapes and surface properties of microchannels to regulate and separate fluids (Gao et al., 2019), (Girault et al., 2017).

Advances in microfluidics have enabled the fabrication of structures at the length scale of individual cells, and in recent years, many types of microscale structures have been developed on microfluidic platforms to study the deformability of single cells. Table 2 below summarizes the various custom microfabricated structures used to measure single-cell deformability, as well as the parameters used to characterize intrinsic cellular deformability.

**TABLE 2:** Microfluidic devices for studying single cell deformability.

Cell type	Microfluidic devices	Parameter Measurement	References
Red blood cells (from newborn reticulocyte to senescent erythrocyte)	Wedge-shaped channel	Area, volume, Hemoglobin	Gifford et al. (2006)
Leukemic cells	Capillary networks	Single-cell transit times	Rosenbluth et al. (2008)
Plasmodium falciparum-infected red blood cells	Wedge shape capillary	Cell surface area and volume, membrane viscoelasticity and cytoplasmic viscosity	Herricks et al. (2009)
Red blood cells	Hyperbolic converging microchannel	Deformation Index (under the extensional flow field.)	Lee et al. (2009)
Human Leukocyte	Capillary structure	Single-cell transit time	Gabriele et al. (2010)
MC-3T3 osteoblast cells	Microfluidic device based on lateral patch-clamping and micropipette aspiration	Young’s model calculated using elastic solid model	Chen et al. (2011a)

### 2.2 Different approaches of microfluidic

Microfluidics can measure cellular deformation by a variety of methods, including: cellular rheology methods, optical deformation methods and a novel type of microfluidics, real-time deformation cytometry. In rheological methods, cell deformation is measured by applying fluid shear using microchannels in a microfluidic chip, and commonly used methods include pulsed shear and constant shear (Forsyth et al., 2010). Optical methods use two optical techniques, optical tweezers and optical stretching, to identify cellular deformability. (Chen et al., 2011a), (Chen et al., 2011b). Real-time cell denaturation uses shear stress to make the cells in the microfluidic channel deform and a high-speed camera to capture images of the cells to identify and analyze their morphology in real-time. The applications of these three methods are summarized below.

#### 2.2.1 Cellular rheology measurement

The cellular rheology measurement methods utilize microfluidics to place individual or multiple cells in a microchannel and sense their deformation in the fluid by applying fluid pressure, to gather information on the mechanical properties of the cells (Shakeri et al., 2021). This method provides highly resolved quantitative measurements of the physical properties of individual cells, which are important for understanding the mechanical properties of cells and the onset and development of related diseases. The key to this method is designing suitable microchannels, which can be produced using methods such as soft lithography or 3D printing, with various sizes and shapes (Razavi Bazaz et al., 2020), (Amin et al., 2016). Before conducting the experiment, cells need to be injected into the microchannels, which can be done manually through individual cell injection or automatically using microfluidics. Next, pressure is applied using a pressure pump or pneumatic pressure to induce cell deformation in the microchannels. By observing cell deformation under different fluid pressures, mechanical parameters such as the elastic modulus and viscosity of the cells can be obtained.

This type of method uses the pressure required to contract a deformed cell at the microscale to measure the deformability of a single cell. This technique is similar in principle to micropipette aspiration but is simpler to perform, less prone to errors, and requires less equipment and technical skill. The ability of this mechanism to measure the degree of cellular deformation was initially validated by testing neutrophils and showed results and measurement accuracy similar to micropipette aspiration. This method has also been used to study the deformability of human erythrocytes, particularly the reduced deformability of erythrocytes parasitized by Plasmodium falciparum. (Lazari et al., 2020).

**FIGURE 1:**
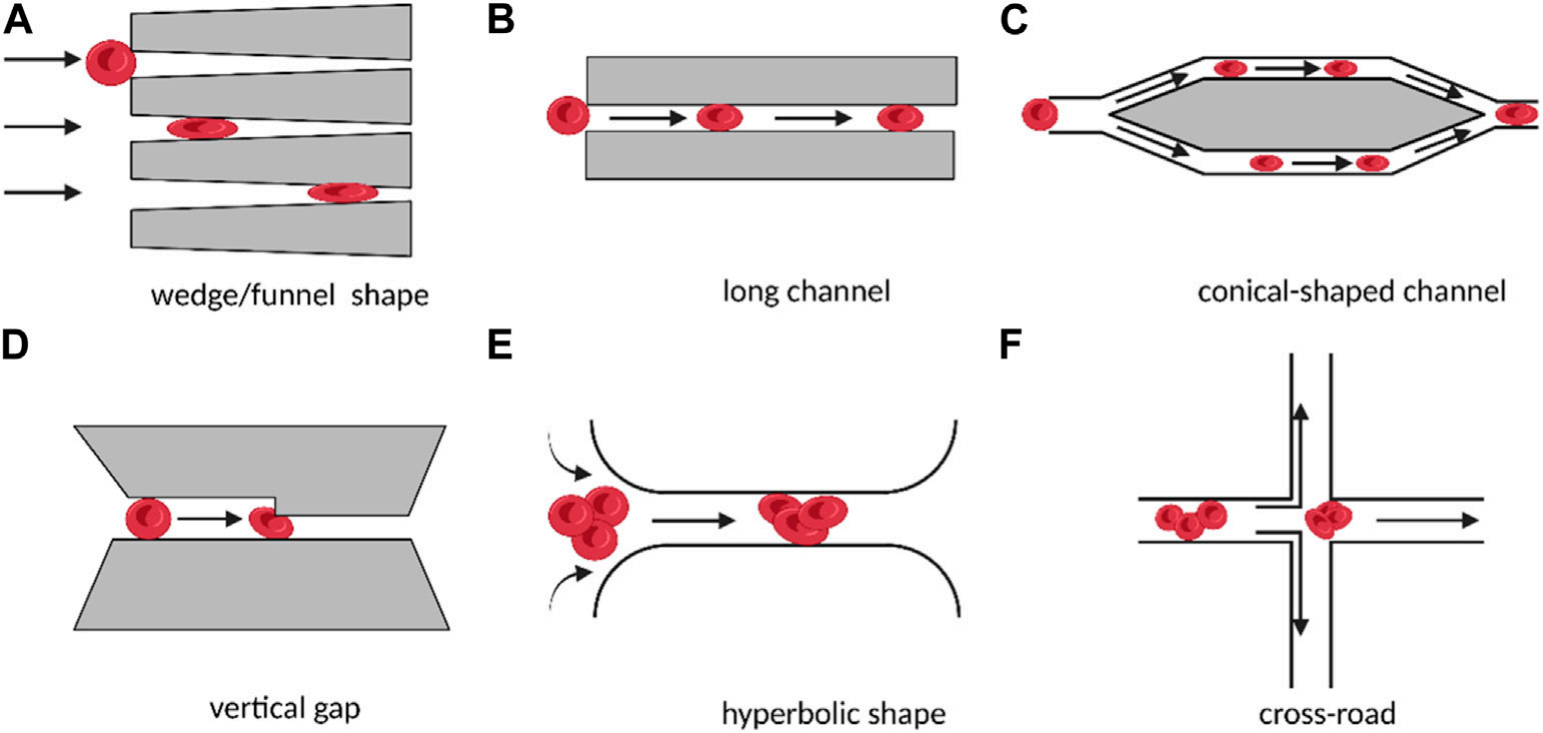
Schematic diagram of different micro-scale structures used for measuring cell deformation in cellular rheology methods. The direct contact between cells and microchannels under the influence of fluid shear stress leads to the deformation of individual suspended cells **(A–D)**. The target cell is located in the converging streamline at the center of the flow, without direct contact with the microstructure, resulting in the deformation of individual suspended cells **(E, F)**. (Created with BioRender.com).

As shown in Figures 1A–D, microstructures such as funnel filters, gap-shaped constrictions, and long conical or wedge-shaped channels have been developed to mimic the internal movement of red blood cells in capillaries (Rosenbluth et al., 2008), (Faustino et al., 2019). These microstructures use specific parameters to describe the deformability of the cell, such as the transit time of the cell during contraction, its minimum cylindrical diameter (determined by measuring its volume and surface area), or its behavior under flow shear stress. Several methods have been used to examine the deformability of blood cells in both healthy and pathological conditions, including red and white blood cells, malaria (red blood cells parasitized by Plasmodium falciparum), sepsis (Lee et al., 2009), and leukocyte sludge. Similar structures have also been found in studies of cancer cell stiffness using metrics such as transit/passage time, entry time (time required for cells to squeeze through microchannels), and transit velocity.

In addition to the direct contact between the cell and the microstructure, microfluidics can also create converging streamlines to deform cells by placing the target cell in the middle of two streamlines (Lee et al., 2009). To produce an extensional flow that results in cell deformation, hyperbolic and cross-road shape structures were designed, as shown in Figures 1E, F. In these designs, the deformability of single cells was expressed and compared using the deformation index, which was defined as the ratio of both axes of the cross-sectional area of a deformed cell.

The microfluidic devices discussed above have several benefits over conventional approaches. These methods streamline the experimental procedure by integrating useful elements, such as cell introduction and capture, into a single microfluidic chip (Zhang et al., 2021). As cells and microstructures are enclosed on the same channel layer, the process is simplified and the step of aligning cells with structures is eliminated. However, the measurement results obtained from the microfluidic devices, such as transit time, velocity, and deformation index, are not highly sensitive. In other words, they rely on the presence of microscopes and cameras to observe and document cell behavior and to simultaneously evaluate cell movement and shape changes. The minimum length step that can be detected by conventional microscopes is approximately 0.1–0.3 μm/pixel, and this resolution is insufficient to provide highly sensitive results (Shi et al., 2021). Therefore, it is essential to develop a new microfluidic method with more precise output measurements.

#### 2.2.2 Optical deformation measurement method

Optical tweezers and optical stretching are two main optical techniques used to identify single-cell deformations. Optical tweezers use a focused laser beam to manipulate two tiny silicon beads immobilized on a single cell (Niculescu et al., 2021). The cells are deformed by extending the distance between the two focal points. The advantage of this technique is that the cells are not directly exposed to the intense focused light, which reduces optical damage to the cells (Chang et al., 2018). However, the technique has a low measurement yield and requires complex instrumentation. On the other hand, optical stretching uses an optical trap based on two Gaussian intensity distributions that can capture cells in suspension and stretch them *in situ*. During optical stretching, radiation damage to the target cells is negligible because the laser beam is divergent and unfocused. Both methods measure the mechanical properties of cells without contacting them, but optical stretching measures the mechanical properties of cells while optical tweezers measure only the local mechanical properties of cells. Additionally, by using the laser heat generated by optical stretching, it is possible to study how heat affects the mechanical properties of the cell.

**FIGURE 2:**
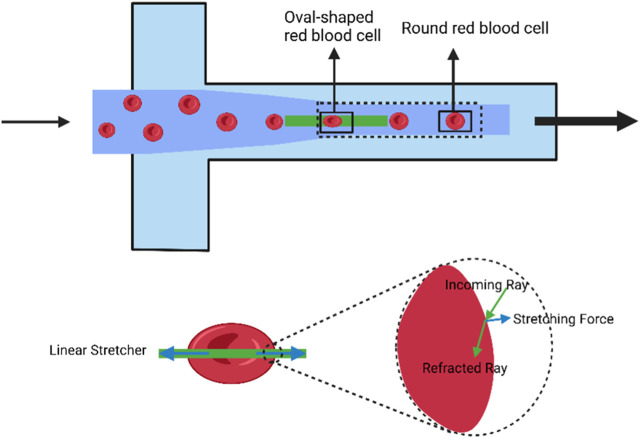
Schematic diagram of the deformation induced in individual cells using the optical stretch deformation cytometer. The optical stretcher captures cells in suspension within an optical trap and stretches them *in situ*. Cells transition from a stretched state to a relaxed state within a rectangular region, changing their shape from elliptical to circular. The solid box represents the region where cell stretching and relaxation occur, and is used to calculate deformation parameters for red blood cells under flow. (Created with BioRender.com).

Optical stretch deformation cytometry enables the assessment of cell deformability in numerous types of cells, including erythrocytes, leukocytes, fibrillogenic cells, and cancer cells. This method is widely used to distinguish between healthy, tumor-causing, and metastatic cells and to investigate how different therapies impact cell deformability. For example, optical stretchers can differentiate the deformation patterns of normal erythrocytes, those treated with diamine and glutaraldehyde, and those combined with neutrophils (Chen et al., 2023). Cell deformation can be determined by comparing the relaxed state of the cell as it exits the trap with its stretched state as it enters the stretched zone (as shown in Figure 2). The use of a microfluidic optical stretcher can also identify malignant fibroblasts. Nava et al. employed acoustic prefocusing and optical stretching to increase the throughput of a microfluidic device (Yoshida et al., 2019). In mouse fibroblast stretching experiments, the integrated device’s stretch throughput increased by 75%. Optical stretchers can be used to distinguish between cells from healthy and cancer patients as well as various types of invasive cancer cells for cancer cell examination (Chen et al., 2023).

#### 2.2.3 Real-time deformability cytometry measurement method

Real-time deformability cytometry is a method for measuring the deformation behavior of cells in a fluidic environment in real-time, using technologies such as microfluidic chips, optical microscopy, and associated data analysis software (Otto et al., 2015), (Toepfner et al., 2018). The method is fast and sensitive, allowing the distribution of size, shape, and deformability of thousands of cells to be assessed in seconds. This method can track and define in more detail the changes in morphological and biomechanical properties of cells during initiation and de-initiation (Bashant et al., 2019). The basic principle of the technique is that cells flow through microfluidic channels in a viscous carrier solution, and pressure gradients and shear stresses deform these cells. A high-speed camera captures an image of each cell as it passes through the microfluidic channel, identifies and analyzes the cell profile in real-time (Mietke et al., 2015). Cell size, deformation, and surface smoothness can be quickly and easily quantified continuously at measurement rates of up to 1,000 cells/second to create RT-DC profiles of a broader range of cell parameters. The brightness of the cell images combined with the area data is sufficient to identify erythrocytes, lymphocytes, monocytes, neutrophils, eosinophils, and basophils in human “whole blood” samples, allowing for greater characterization of all major immune cell types simultaneously (Bashant et al., 2019). Only a few microliters of blood are required to generate such RT-DC profiles, and these findings can be independently verified in isolated cell populations. In summary, RT-DC provides a fast and accurate method to analyze cell size, deformation, and shape. It combines speed, ease of use, and integrity that were not previously available (Rosendahl et al., 2018). Real-time cellular denaturation can be used in many applications such as cellular biomechanics studies, cellular oncology, and drug screening. Compared to traditional cellular mechanics testing techniques, real-time cellular denaturation offers the advantages of ease of operation, high throughput, high sensitivity, and non-invasiveness (Girardo et al., 2018).

**FIGURE 3:**
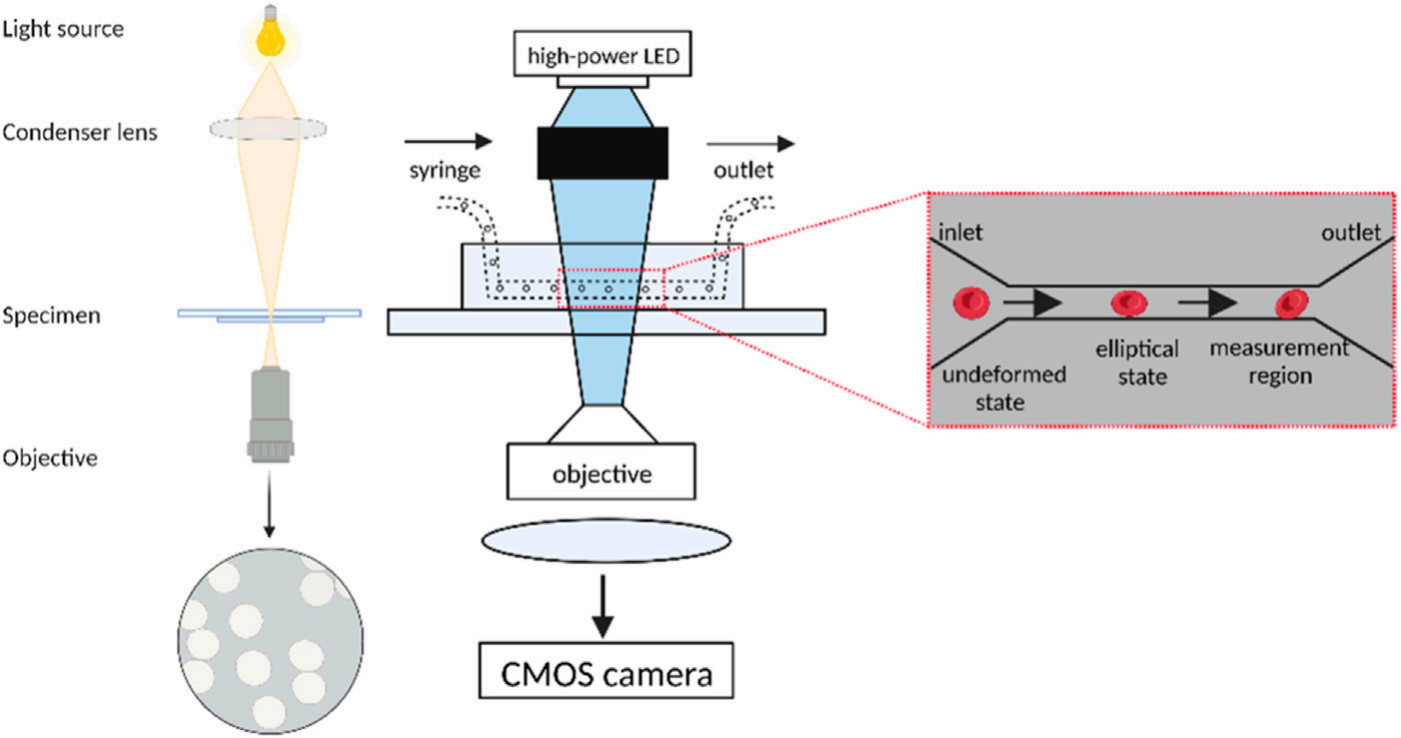
Schematic diagram of the Real-time deformability cytometry. The undeformed cells enter the narrow microfluidic channel in an elliptical shape and undergo deformation under the influence of pressure gradients and shear stress. The cells are captured by a high-speed camera at the end of the channel for real-time identification and analysis of the cells as well as contour measurement using data analysis software. (Created with BioRender.com).

In the study by Bashant et al. (2019), real-time deformability cytometry was employed to investigate neutrophils. The neutrophils were gently mixed and then resuspended in Cell Carrier solution, which is a solution based on 1×PBS buffer containing 0.5% methylcellulose, at a concentration of 2.5 × 107 cells/mL. The cell suspension was pumped into a 1 mL syringe and placed into the gas pedal syringe pump, which was then extended for inverted microscopy. The Flic20 PDMS microfluidic chip was used for sample intake, and the chip’s reservoir layer was connected via a square measuring channel with a 20 × 20 μm^2^ cross-section. Another syringe filled with Cell Carrier buffer and free of cells was placed in the syringe pump and connected to the sheath inlet of the microfluidic chip. RT-DC measurements were collected at a cell flow rate of 0.12 μL/s, with 0.03 μL/s sample flow and 0.09 μL/s sheath flow. An open-gating strategy was used for isolated cell populations, whereas for whole blood measurements, 50 μL of blood was diluted into 950 μL of Cell Carrier buffer, and cell gates of 5–16 μm parallel to the flow direction and 5–20 μm perpendicular to the flow direction were applied. This was sufficient to exclude single erythrocytes and double erythrocytes. Cell images were captured by an inverted microscope combined with a high-speed CMOS camera at a frame rate of 2000 fps as the cells reached the end of the constriction channel. The structure of the RT-DC microfluidic chip is shown in Figure 3 below.

Real-time deformability cytometry (RT-DC) is an emerging microfluidic technique that allows for the capture and evaluation of the morphology and rheology of up to 1,000 cells per second in a narrow channel. This method has many possible applications in biology and biomedical research. Cells are deformed without fluid mechanical contact, and segmented images of the cells are stored quantitatively in real time without additional processing or staining procedures. These images can be used for further analysis. This technique is particularly attractive since it offers the possibility to characterize cells without any external markers and to sensitively detect physiological and pathological changes in cell function (Herbig et al., 2018). RT-DC is sensitive to changes in the cytoskeleton and can, for example, show differences in the temporal phase of the cell cycle, identify different subpopulations in whole blood, and study mechanical sclerosis of cells entering a dormant state (Mokbel et al., 2017).

Table 3 summarizes and compares the different microfluidic techniques, including the principles, applicable objects, and their advantages and limitations.

**TABLE 3:** Summary and comparison of microfluidic technologies.

Approach	Fundamental principle	Applicable objects	Advantages	Limitations
Cellular rheology measurement	The deformation of cells in microchannels was measured by hydrodynamics	Blood cells, liver cells and lung cells	Fast measurement speed, high precision and repeatability	The cells need to be treated to obtain a certain cell concentration, and the cells are subject to shear forces, resulting in measurement errors
Optical deformation measurement	Stretching cells through two interacting light beams in a microfluidic channel to produce cell deformation	Red blood cells, White blood cells, stem cells, fibroblasts and cancer cells	Fast measurement speed, low sample consumption, low experimental cost, automatable and highly controllable	Multiple parameters cannot be measured simultaneously, special optical equipment is required, and assumptions about cell shape may affect the accuracy of the measurement results
Real-time deformability cytometry measurement method	The deformation properties of cells are measured and analyzed by applying different levels of shear force to induce cell deformation and recording the deformation process by a fast imaging system	Cancer cells, immune cells, and red blood cells	Real-time detection, high-throughput measurement, no damage to cells	Shear force may have an impact on the physiological functions of cells, leading to errors in measurement results, and the high cost of equipment and operation limits its popularity in practical applications

## 3 Applications in clinical diagnosis

### 3.1 Applications of microfluidics in blood cell deformation studies

Red blood cells are the most common type of cell in the blood and their main role is to absorb and transport oxygen from the alveoli to the tissues and organs of the body by carrying hemoglobin (Yoshida et al., 2019). The morphology and deformability of red blood cells allow them to flow unimpeded through the blood vessels, thus ensuring the supply of oxygen to all parts of the body and the removal of carbon dioxide waste. Red blood cells must be highly deformable in order to flow easily through capillary networks and sinusoidal pores that are smaller than their own diameter. (Quinn et al., 2011). In certain pathological conditions, red blood cell deformability is reduced, causing an increase in blood viscosity, peripheral resistance, and impaired microcirculation. (McMahon, 2019). Therefore, studying and monitoring the deformability of erythrocytes is important for the diagnosis and treatment of many diseases (Skalak and Branemark, 1969). In order to investigate the flow behavior and deformation characteristics of erythrocytes in capillaries, many types of microfluidic devices, such as single-channel and multi-channel, have been constructed. The single-channel microfluidic chip consists of a tiny channel and microstructure with a relatively simple structure that allows accurate detection of the flow and deformation properties of individual blood cells in capillary channels. Compared to multi-channel microfluidic chips, molecular and cellular behavior within the microfluidic fluid can be detected more accurately and experiments can be completed in a shorter time frame. Multichannel microfluidic chips, also known as integrated microchannel chips, have multiple tiny channels and microstructures, so that multiple samples can be processed in parallel on the same chip, offering the advantages of high integration, high throughput, high accuracy and fast response. The multidimensional grid layout of multi-channel microfluidic chips can simulate the intricate microcirculatory environment and vascular branching network of blood flow, which can be used to study processes such as microvascular occlusion and thrombosis.

#### 3.1.1 Single-channel microfluidic chip

**FIGURE 4:**
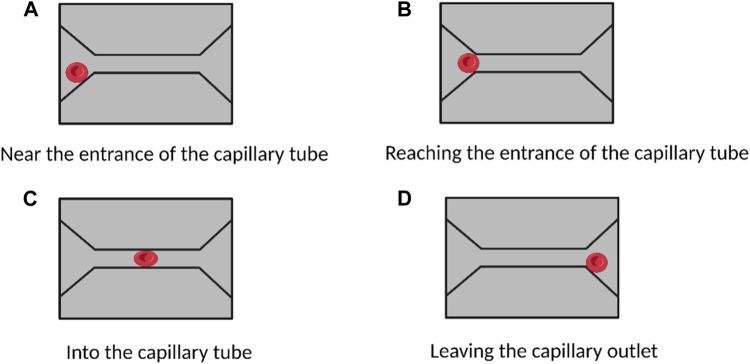
Study of red blood cells flow and deformation in capillaries based on a single-channel microfluidic device. **(A–D)** show the deformation of red blood cells when they are in different areas of the capillaries. No morphological changes occurred when the cells approached and reached the capillary entrance. When entering the capillaries, the cells undergo a large morphological change. After passing through the capillaries, the cells return to their natural state. (Created with BioRender.com).

Single-channel microfluidic chips enable high-sensitivity studies of intracapillary flow in red blood cells, allowing for direct observation of dynamic and discontinuous processes at the cellular and subcellular levels. This technology can improve our understanding of various erythrocyte diseases. For example, Quinn et al. used microfluidic chip technology to quantitatively study erythrocyte flow patterns in capillaries (Quinn et al., 2011). By varying the pressure gradient at the entrance and exit of the channel, they were able to measure the flow rate of erythrocytes through capillaries at different pressure drops and found that the flow rate varied almost linearly with the pressure gradient. Li et al. developed a new single-channel microfluidic device to study the flow characteristics of erythrocytes in capillary blood flow (Li et al., 2007) (Figure 4). They observed that when erythrocytes squeezed into the narrow capillary channel (only 4 μm in size), they underwent considerable deformation (Figure 4C) but quickly returned to their natural disk-like structure after passage (Figure 4D). Thus, subcellular-level modeling and single-cell microfluidic chip-based experiments can help us better understand erythrocyte flow patterns in capillaries.

**FIGURE 5:**
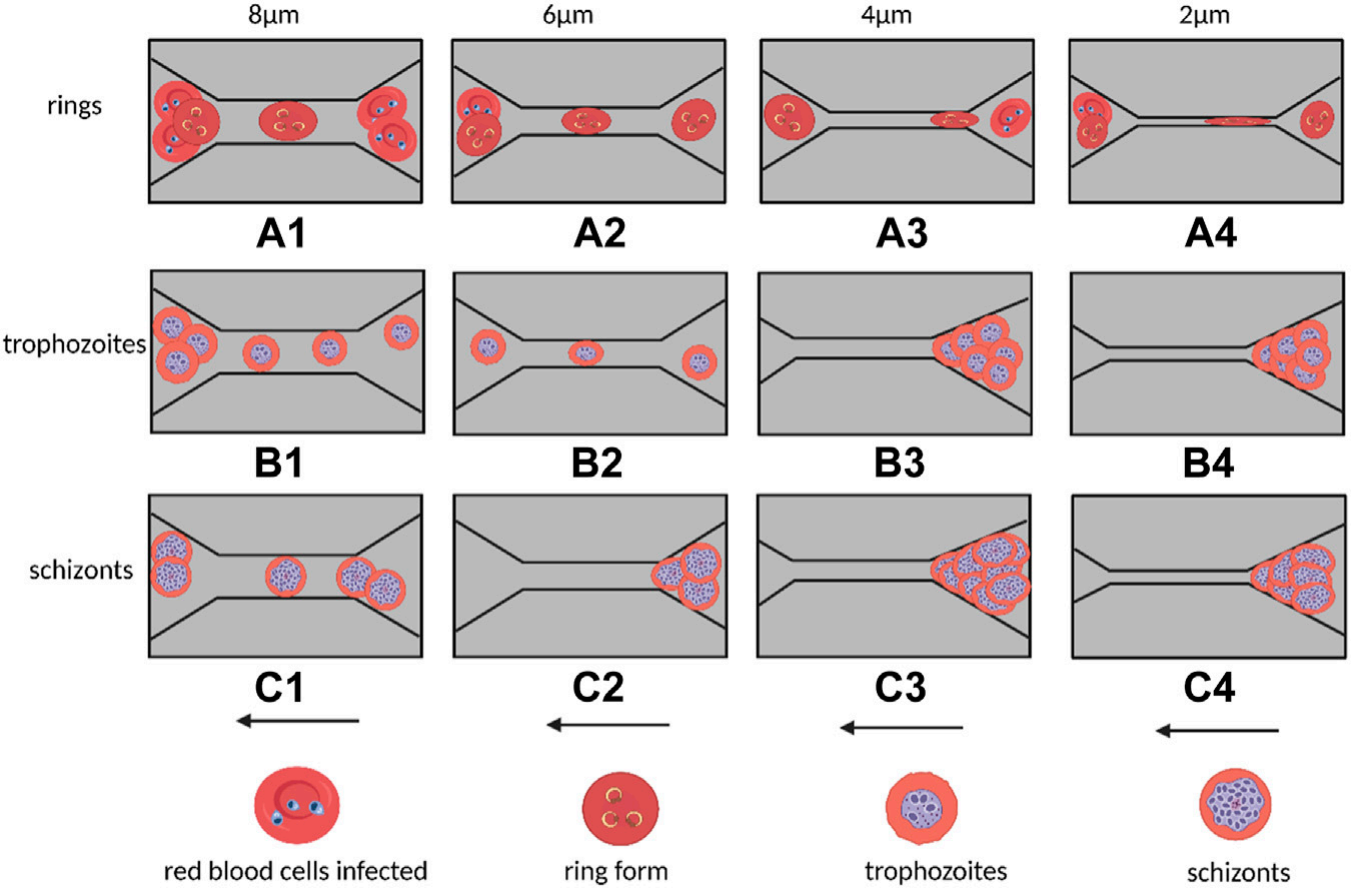
Flow of malaria-infected erythrocytes within capillaries of different sizes in a single-channel microfluidic device and blockage behavior. The widths of the capillaries from left to right are 8, 6, 4, and 2 μm. Arrows indicate the direction of cell flow. **(A1–A4)** indicates that the cyclosomal erythrocytes at the early stage of infection can pass through the capillaries smoothly; **(B1–B2)** represents that the erythrocytes at the end of infection can only pass through the 8 and 6 μm capillaries, and **(B3–B4)** represents that the erythrocytes at the end of infection cannot pass through the capillaries and cause blockage of the microchannels. **(C1)** Indicates that the erythrocytes at the Schizont stage can only pass through capillaries of 8 μm but not capillaries of 6 μm and below **(C2–C4)** (Created with BioRender.com).

Erythrocytic hematologic disorders are a group of diseases that affect the morphology, number, or function of red blood cells. Recent studies have shown that the generation and development of many hematologic diseases are closely related to the membrane structure and deformation characteristics of red blood cells. Today, microfluidic microarrays can be used to assess the deformation characteristics of diseased red blood cells to obtain critical information about the clinical status of hematologic diseases and the effectiveness of drug therapy. For example, Shelby et al. (2003) developed a single-channel microfluidic chip to analyze the deformation properties of erythrocytes caused by Plasmodium falciparum infection, (Guo et al., 2012), (Guo et al., 2014). They extracted erythrocytes with different levels of infection from the patient’s blood, diluted the samples with a viscous solution, and then passed the diluted samples through small rectangular capillary microtubes of different widths (Figure 5). The shape and stiffness of these infected erythrocytes influenced the way they flowed within the microchannels. The experimental results showed that ring-like body (Ring) erythrocytes at the beginning of infection could pass normally through the capillary microtubes (Figures 5A1–A4, Trophozoite red blood cells could pass through micro ducts of wider sizes (6 and 8 μm) (Figures 5B1, B2), but not through narrower (2 and 4 μm) microtubes (Figures 5B3, B4), while erythrocytes in the Schizont stage had significantly weaker deformation properties and could only pass through capillary tubes of 8 μm (Figure 5(c1)), but not through capillary tubes of 6 μm and below (Figures 5C2–C4), which may even cause blockage of capillaries.

#### 3.1.2 Multi-channel microfluidic chips

Due to the very small size of blood cells, it is difficult to analyze and detect them accurately using conventional assays. Therefore, microfluidic devices have emerged as a viable method for blood cell performance analysis. (Liu et al., 2022), (Li et al., 2017a), (Qiang et al., 2017). However, due to the small size of individual blood cells, microfluidic devices can only be used to test microscopic blood samples. (Marrella et al., 2021). As a result, in most cases, the number of blood cells suffering from certain viral or bacterial infections or other diseases is very small and can only be diagnosed and studied by detecting a small number of blood cells in a microscopic blood sample. Therefore, how to analyze and detect infected red blood cells in very small amounts from micro blood samples is an urgent problem. To this end, multi-channel microfluidic chips have been designed for direct mechanical analysis and deformation property measurement of blood cells at high throughput and high sensitivity (Ma et al., 2021), (Qiang et al., 2019). Meanwhile, the multidimensional network structure of the microfluidic chip can be connected into a network, which can simulate the complex geometry and more realistic blood flow microcirculation environment. Currently, multichannel microfluidic chips have been used to study the deformation, aggregation and microthrombosis of blood cells in health and disease (Li et al., 2017b). This study summarizes the study of the flow behavior of malaria-infected erythrocytes in capillaries based on multichannel microfluidic devices.

**FIGURE 6:**
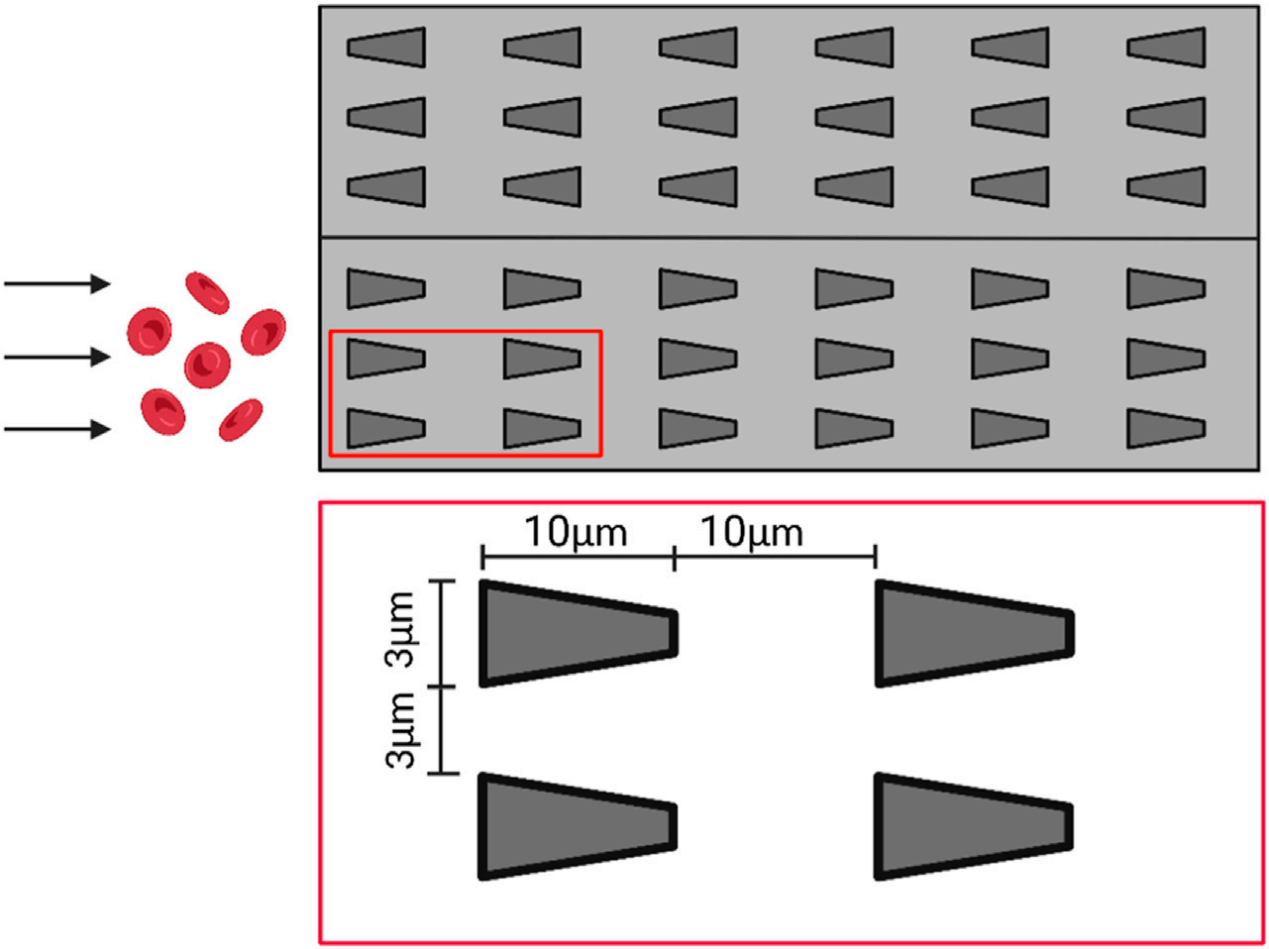
Schematic diagram of the multi-channel microfluidic device. The microfluidic channel contains several side-by-side arrays of microtubules with a width of only 3 μm, which deform the red blood cells as they flow through the arrays (Created with BioRender.com).

Bow et al. designed a multichannel microfluidic device capable of performing direct deformation performance assays on infected red blood cells from blood samples containing only a very small number of malaria-infected red blood cells (Bow et al., 2011). The microfluidic device has multiple side-by-side arrays of microtubules that are only 3 μm wide (as shown in Figure 6). As the red blood cells flow within the microfluidic channels, they must deform to flow through the microtubule arrays. The results showed that malaria-infected erythrocytes had more difficulty squeezing into the narrow microchannels and flowed more slowly within the microfluidic tracts than healthy erythrocytes, indicating that infected erythrocytes had significantly reduced deformation properties. Additionally, the researchers found that the shape of the microtubule entrance (constricted entrance and divergent entrance) also had a significant effect on the flow behavior of erythrocytes. Infected erythrocytes were more likely to enter the constricted-entry microtubule, and their flow velocity within the microfluidic channel was significantly increased compared to the results of the divergent-entry microtubule. Therefore, this microfluidic chip device could better analyze the flow behavior of infected red blood cells and help medical practitioners to better diagnose diseases. The device was designed to solve the problem of detecting infected red blood cells in microscopic blood samples where the number is so small that a single-channel microfluidic chip cannot meet the need. The device could detect multiple blood cells simultaneously, thus greatly improving the accuracy and efficiency of the test.

### 3.2 Applications of microfluidics in stem cell deformation studies

Stem cells are a type of cell that has the ability to self-renew and differentiate into multiple types of cells, and are often referred to as “master cells” (Wan et al., 2021). They play a crucial role in tissue regeneration and repair, and have promising applications in regenerative medicine. Deformation of stem cells can significantly affect their behavior and differentiation potential (Deng et al., 2017). For instance, deformation can alter their morphology, cytoskeletal structure, and gene expression profiles, ultimately affecting their fate of differentiation. Microfluidic cell deformation measurements have multiple applications in stem cell research, including the characterization of stem cell mechanical properties such as stiffness and deformability, which can provide information about their differentiation potential and tissue integration ability (Huang et al., 2019). Microfluidic devices can apply controlled mechanical forces to stem cells, such as tensile, compressive, and shear stress, inducing deformation and changing their mechanical properties. Moreover, microfluidic technology provides a platform for high-throughput analysis of stem cell deformation under various physical and chemical stimuli, elucidating potential mechanisms and pathways involved in deformation. Microfluidic cell deformation measurements can also be used to study stem cell differentiation into different cell types (Zhou et al., 2021). Stem cells typically undergo a series of shape changes during differentiation, which can be measured using microfluidic devices. By analyzing stem cell deformation in response to different cues, such as growth factors or mechanical stimuli, researchers can gain insights into the mechanisms of stem cell differentiation and optimize differentiation protocols. Microfluidic devices can also be designed to mimic *in vivo* microenvironments, such as extracellular matrix stiffness and topography, which are crucial for stem cell behavior (Menachery et al., 2017). The study of stem cell deformation using microfluidic technology is of significant importance to regenerative medicine and tissue engineering. By understanding stem cell responses to mechanical forces and deformation, researchers can better control their differentiation fate and increase their therapeutic potential (Coles et al., 2021). Furthermore, microfluidic technology can be utilized to screen drugs and growth factors that can regulate stem cell deformation and differentiation, ultimately leading to the development of novel stem cell-based therapies for various diseases and injuries (Hodgson et al., 2017), (Sibbitts et al., 2018). In summary, microfluidic cell deformation measurement provides a powerful tool for studying stem cell mechanical properties, understanding the mechanisms of stem cell differentiation, and developing new stem cell-based therapies.

A population of pluripotent colony-forming cells that express membrane markers when cultivated on flat polystyrene tissue culture surfaces is known as human mesenchymal stem cells (hMSCs). Beijer et al. used micro-scale specified surface topography as a model to characterize the phenotypic changes of hMSCs adjusting to a new environment. When the cells were exposed to surface topography, they underwent considerable changes in morphology, including cytoplasm and nucleus shrinkage, a general decrease in cell metabolism, and a sluggish cell cycle progression that led to lower cell proliferation rates. This adaptive phenotype may reduce the likelihood of cellular process dysfunction, thereby lowering the number of metabolic stressors. Consequently, topography-enhanced substrates can be used as a model system to investigate the most fundamental cellular processes related to cancer and aging (Beijer et al., 2019). To improve the therapeutic effect of cell-based regeneration or repair, Jin et al. developed a microfluidic device for studying cellular responses and response pathways in rat bone mesenchymal stem cells (BMSCs) cultured on microspheres of predetermined curvature (Jin et al., 2022). Circulating tumor stem cells have attracted attention in recent years because of their higher tumorigenicity compared to non-stem cell-like circulating tumor cells (Cho et al., 2021). Cell mechanics-based microfluidic sorting systems provide an efficient and specific platform for studying stem cell-like properties based on cell deformability and cell-matrix adhesion properties. Therefore, Jia et al. created a microfluidic tandem mechanical sorting system for isolating circulating tumor stem cells. The resulting deformable and low-adherent cancer cells displayed improved stem cell-like properties *in vitro* and *in vivo* compared to single device isolation, including higher stemness and metastatic capacity (Jia et al., 2021). Menachery et al. investigated the relationship between cell size and deformability using a passive size separation technique based on microfluidics, and then optimized the design and flow rate to increase the recovery and purity of stem cell separation (Menachery et al., 2017). Microfluidics can provide high-throughput and high-precision methods for cell analysis and manipulation, making it a powerful tool for studying stem cell deformation.

### 3.3 Applications of microfluidics in cancer cell deformation

Cancer is currently one of the most predominant non-communicable diseases in humans. In cancer, cells lose their normal control mechanisms for growth and differentiation, leading to unrestricted proliferation and invasion of surrounding tissues (Chaudhuri et al., 2016). The deformation of cancer cells may result in evasion of immune surveillance, resistance to chemotherapy drugs, and increased invasive capacity, making the study of cancer cell deformation crucial for cancer treatment and prevention (Roberts et al., 2021). With improved understanding of cancer biology and advances in microfluidics, microfluidics has been validated for cancer detection, diagnosis, and treatment (Armistead et al., 2019). Microfluidic cell deformation measurements have multiple applications in cancer research. One of the major applications is in the study of cancer cell migration and invasion. Cancer cells often have altered mechanical properties that enable them to migrate and invade surrounding tissues. Microfluidic devices can be used to induce controlled deformation of cancer cells and measure their response, providing insight into the underlying mechanisms of cancer cell migration and invasion. In addition, microfluidic cell deformation measurements can assess the effectiveness of anti-cancer drugs. Cancer cells often exhibit altered mechanical properties that make them more resistant to drugs (Shaw Bagnall et al., 2015). By measuring the deformation of cancer cells in response to drug treatment, researchers can evaluate the effectiveness of different drugs and optimize treatment strategies. Microfluidic devices can also be used to study the interactions between cancer cells and the surrounding microenvironment, such as the extracellular matrix or immune cells (Allard et al., 2004). By measuring the deformation of cancer cells in response to different microenvironmental cues, researchers can gain insight into how the microenvironment affects cancer cell behavior and develop new approaches to target cancer. Microfluidics can also provide new ideas and approaches for developing anti-cancer therapies, such as screening compounds and targets that interfere with cancer cell deformation (Castro-Giner and Aceto, 2020). In summary, the application of microfluidics in the study of cancer cell deformation has significant importance and broad development prospects.

During metastasis, cancer cells leave the primary tumor, enter the circulatory system, and infiltrate new tissues. Cells must be able to efficiently alter their shape in order to squeeze through the small interstices of the extracellular matrix or to infiltrate the blood or lymphatic system as they migrate across the diverse environments they encounter. Circulating tumor cells (CTCs) are isolated tumor cells that disseminate from the site of disease in metastatic or primary cancers (Liu et al., 2013), (Burinaru et al., 2018). They can be used as a diagnostic tool to assess the severity of the disease, track the success of treatment, and serve as a stand-alone prognostic factor (Wei et al., 2021). A significant biophysical indicator of a cancer cell’s ability to metastasize, adhesion strength is intimately linked to several metastatic processes (TruongVo et al., 2017). There is growing evidence that cancer cells are soft, and that softer cells are more aggressive when they metastasize to other organs, including bone. The strong deformability of cancer cells, which enables them to move in a 3D-restricted environment, is linked to their aggressiveness (Wolf et al., 2007). For instance, human pancreatic cancer cells have been observed to dramatically deform during migration in large-scale cultures when their protein hydrolytic activity is blocked (Huang et al., 2011). The cytoskeleton and other organelles undergo significant rearrangement in tandem with the distorted movement of cancer cells. Cancer cells are more resilient than non-cancerous cells and modify their migratory processes to adapt to various situations. Therefore, a deeper comprehension of cell migratory mechanisms is necessary for therapeutic efforts to immobilize cancer cells by inhibiting pertinent signaling pathways. Size-based microfluidic devices are widely used for the isolation of circulating tumor cells, and these devices can sense the different diameters of cancer cells, red blood cells, and white blood cells (Huang et al., 2011). To classify different cancer cells of similar size, researchers use constriction channels and study the kinetics of the cells, often with other methods sensitive to biomechanical and bioelectrical properties as well, to achieve cell identification (Ren et al., 2017).

**FIGURE 7:**
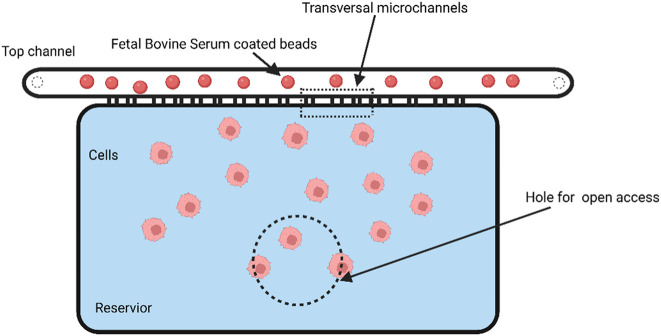
Schematic of an open-channel microfluidic device for studying the cell deformation during migration in a confined environment. The dashed line indicates the area that is perforated to provide open access (Created with BioRender.com).

Using metastatic breast cancer cell lines, Michael Mak and David Erikson developed an automated micropipette device that enables the recording of cellular deformation and relaxation. Their device has parallel microchannels with several microcontractions for each cell that will deform the cells multiple times. Their research showed that tumor cells move more swiftly through subsequent constrictions after navigating the initial one. According to this finding, tumor cells deform when they infiltrate the extracellular matrix with subnuclear pore size and may spread more easily (Mak and Erickson, 2013). Zhang et al. reported a microfluidic approach to enrich physically deformed cells by mechanical manipulation of an artificial microbarrier, driven by hydrodynamic forces, where flexible cells or cells with a high metastatic propensity can pass through the microbarrier and leave the separation device, while rigid cells remain trapped. They demonstrated a mixture of two breast cancer cell types with different deformability and metastatic potential, and the isolation of heterogeneous breast cancer cell lines into enriched flexible and rigid subpopulations. This flexible phenotype was associated with overexpression of multiple genes involved in cancer cell motility and metastasis, as well as higher efficiency of mammosphere formation. The experimental results support the relationship between tumor initiation capacity and cellular deformability and demonstrate that tumor-initiating cells are less differentiated in terms of cellular biomechanics (Zhang et al., 2012). In order to explore how normal and cancer cells behave in small spaces, TruongVo et al. built and constructed a microfluidic channel that allowed them to distinguish between the two types of cells by observing how they rest and move across the space. Softer cancer cells move through the channel more quickly and farther than normal cells due to their higher deformability (Chaw et al., 2007). Chaw et al. set up a multi-step microfluidic device for the study of primary tumor cell deformation and extravasation. The deformation, biology, and migration ability of various tumor cell lines were quantified using this device. After deformation, cell viability was significantly reduced, and the number of ploidy was simultaneously increased, indicating a change in their biological capacity. However, cell deformation did not significantly reduce cell motility (Mak and Erickson, 2013). Malboubi et al. created a microfluidic device (as shown in Figure 7) to research how cancer cells can modify the form of their nuclei and cells as they move through tiny openings. The open, simple-to-use gadget enables the concurrent investigation of the impact of various degrees of space restriction on cell activity and morphology. The findings demonstrate that cells have a threshold cross-section below which they cannot penetrate microchannels and that their capacity to migrate into microscopic gaps is reduced as cellular limitation increases (Malboubi et al., 2015).

Microfluidics can be employed to measure the mechanical properties of cells, including their elasticity and mobility (Lange et al., 2015). Additionally, microfluidic platforms can facilitate label-free cell sorting, which involves using image-based cell sorting methods that incorporate machine learning approaches and deep neural networks (DNN) to transfer the molecular specificity of conventional cell labeling to label-free sorting. The sorted cells can then be thoroughly analyzed and examined for their proteomic, transcriptomic, or genetic properties and functions, or for applications in regenerative medicine. (Nawaz et al., 2020), (Isozaki et al., 2020), (Zhang et al., 2022).

## 4 Translational impact of microfluidic-based cell deformation measurement

Microfluid-based cell deformation measurement techniques provide an accurate and quantitative way to measure cell deformation, which can be used for disease diagnosis and therapeutic monitoring. Although these methods are primarily used in laboratory Settings, their translational impact holds significant promise for applications in different fields (Mount et al., 2015). The translational impact of microfluid-based cell deformation measurements is that it has the potential to improve patient outcomes by enabling earlier disease detection, personalized treatment plans, and improved monitoring of disease progression. This section aims to discuss the potential translational effects of microfluid-based cell deformation measurements and their implications for biomedical research, clinical diagnosis, and therapy.

First, the translational impact of microfluidics-based cell deformation measurement on biomedical research is remarkable, providing a new technical tool for biomedical research. This measurement method can help researchers analyze cell deformability and thus gain insight into cell mechanics, which can reveal the pathogenesis of diseases such as cancer, cardiovascular diseases and genetic disorders. Researchers can also obtain information about the mechanical properties of cells and their changes in different disease states, which can help identify new biomarkers, therapeutic targets, and the development of personalized therapeutic strategies based on the mechanical properties of cells (LeBleu and Kalluri, 2018). In addition, the technology has an important translational impact in clinical diagnosis. Traditional diagnostic approaches typically rely on molecular markers or imaging techniques that may not capture subtle changes in cellular mechanics. In contrast, microfluidics-based cellular deformation measurements can provide complementary information that enables the detection and monitoring of mechanistically significant diseases such as cancer metastasis, sickle cell disease, and malaria (Liu et al., 2016). The translational impact of this technology also extends to developments in drug development and therapy. By assessing cell mechanics properties, researchers can evaluate the impact of different therapeutic interventions on cell deformability, which in turn can guide the design and optimization of therapeutic strategies, including drug delivery systems, tissue engineering approaches, and personalized medicine. Such measurements can improve drug development efficiency, reduce the use of animal models, and make drug development more reliable and effective (Kovarik et al., 2012). At the same time, cell deformation measurements can be used for drug screening and assessment of drug efficacy, thus shortening the drug development cycle. Finally, one of the advantages of microfluidics is the ability to miniaturize measurement devices and integrate them into portable devices. This means that real-time cell mechanics analysis can be performed rapidly at the side of the patients, thus opening up new possibilities for early disease detection, therapeutic response monitoring, and personalized medicine (Nge et al., 2013). These devices can be deployed in resource-limited settings, providing affordable, accessible diagnostic tools in underdeveloped areas. The translational impact of this technology is potentially of great value in improving healthcare and health management, especially in areas lacking medical resources (Xia et al., 2016). Thus, the translational impact of microfluidics-based cell deformation measurements for Point-of-Care Applications is significant and offers new approaches and possibilities for improving healthcare.

Overall, microfluidics-based cellular deformation measurements have tremendous potential to advance biomedical research, clinical diagnostics, therapeutic and drug development, and point-of-care applications. The translational impact of microfluidics-based cellular deformation measurements is far-reaching and has the potential to revolutionize various fields by improving disease detection, treatment and management, providing a new tool for studying cellular mechanics and its effects in different contexts. Its high-throughput and high-resolution capabilities enable new applications that were previously impossible, leading to new insights and opportunities for innovation.

## 5 Conclusion and outlooks

This paper introduces the background and significance of microfluidic devices for measuring cell deformation. It reviews the applications of different types of microfluidic devices in cell deformation measurement, including passive, active, and hybrid devices. The advantages and disadvantages of various technologies and their applications in biomedical fields are discussed. Moreover, the paper analyzes the advantages and disadvantages of various microfluidic devices for cell deformation measurement and summarizes the applications of microfluidic techniques for measuring blood cell deformation. These approaches take previous results of mechanical studies on specifically isolated blood cells to the level of an application directly in blood, adding a functional dimension to conventional blood analysis.

In addition, the identification and separation efficiency depend on the flow rate and the shape of the microcolumn, and the results obtained in turn can be used to support the need to redesign a more optimized microfluidic structure for accurate separation and measurement of deformation. These studies have revealed that cell deformation is determined by cell stiffness and depends on cell size in relation to microfluidic channel size. The deformation of cells within the channels and the quantification of the mechanical parameters of the cells can be assessed by hydrodynamic and linear elasticity theories. It is anticipated that these new models will be widely used for basic research and medical applications, with direct examples including label-free sorting of hematopoietic stem cells for transplantation or tumor circulating cells.

Measurement of cell deformation by microfluidics is an important research directions in the field of cell biology and biomedicine. Several microfluidics-based methods for cell deformation measurement have emerged, but these methods still have some limitations and challenges. Current microfluidics methods for measuring cell deformation mainly rely on manual visual analysis, which is subjective and error-prone. In the future, introducing technologies such as machine learning and artificial intelligence can build more accurate, automated and intelligent cell deformation measurement methods. In addition, current microfluidic chip designs often only provide a single cell deformation method, such as stretching or compression. In the future, more diverse and adjustable microfluidic chips can be designed in order to study the deformation characteristics of different cell types and different cell states. The deformation behavior of cells in physiological environments is often influenced by a variety of factors, such as mechanical properties, chemical properties, and biomolecular signals. In the future, microfluidics can be considered to combine with bionics to construct more complex and realistic bionic environment simulations to better study the cell deformation mechanisms. The technological development of microfluidics to measure cell deformation has a wide application prospect and potential, and will have more accuracy, automation, diversity and realism in the future development.

Microfluidic techniques to measure cellular deformation are important in clinical applications and can be used to study and diagnose a variety of diseases, such as cancer, cardiovascular diseases, and neurological diseases. In early cancer screening, microfluidics measurement of cell deformation can detect the morphological and deformation properties of cells, which are closely related to the morphology and behavior of tumor cells. Therefore, microfluidics can be used to detect cancer cells in body fluids or circulating tumor cells for early cancer screening. In addition, microfluidics can monitor the response of tumor cells to treatment and thus provide guidance on treatment options. For example, microfluidic chips can be used to simulate the morphology and deformation characteristics of tumor cells before and after treatment, thus assessing the effectiveness of the treatment. Also microfluidics can detect the deformation properties of red and white blood cells, which are closely related to the onset and progression of cardiovascular diseases. Therefore, microfluidics can be used to detect the deformation properties of cells in blood to assist in the diagnosis and treatment of cardiovascular diseases. Microfluidics can also mimic the morphology and deformation properties of nerve cells, thus studying the mechanism and pathophysiology of neurological diseases. For example, microfluidic chips can be used to simulate the morphological and deformation properties of nerve cells, thus studying neurodegenerative diseases and neural reconstruction, among others. In conclusion, the measurement of cell deformation by microfluidic technology has a wide application prospect in clinical applications, which can be used to study and diagnose a variety of diseases and provide more accurate and personalized treatment plans for clinical medicine.

As microfluidic chip technology continues to develop, simulating more complex geometry and more realistic blood flow microenvironments and linking the dynamic changes of blood cell shape with the pathological changes and clinical manifestations of various diseases will be key focus areas and challenges in future work. The future direction in microfluidic measurement of cell deformation is likely to focus on the development of more advanced microfluidic devices that can provide more precise and complex control over cellular deformation. This could include the integration of multiple microfluidic channels or the use of 3D microfabrication techniques to create more complex and sophisticated microfluidic structures. Additionally, there may be an increased focus on developing microfluidic devices that can simultaneously measure multiple aspects of cellular biomechanics, such as cell stiffness, adhesion, and deformation, to provide a more complete understanding of cellular function. Finally, there may be an increased interest in developing microfluidic devices that can be easily integrated with other analytical techniques, such as imaging or mass spectrometry, to provide a more comprehensive analysis of cellular behavior. Overall, the future of microfluidic measurement of cell deformation is likely to focus on developing increasingly sophisticated and integrated devices that can provide a more comprehensive understanding of cellular biomechanics and function. Moreover, machine learning artificial intelligence could be used for prediction, judgment, and optimization processing of microfluidic chip flow channel design in future research work.

Finally, the development of more intelligent microfluidic devices and the combination of multiple technologies for cell deformation measurement are expected to further enhance the value of microfluidic technology in the biomedical field and bring more opportunities and challenges for biomedical research and diagnosis and treatment.
